# Data accuracy in the European Cystic Fibrosis Society Patient Registry: results of an on-site data validation project

**DOI:** 10.1186/s13023-025-04153-w

**Published:** 2025-12-02

**Authors:** Naehrlich Lutz, Fox Alice, Krasnyk Marko, Wollscheid Nadine, Silvia Lorca Mayor, Zolin Anna, Prasad Vibha, ECFS Patient Registry Steering Group

**Affiliations:** 1 Department of Paediatrics, Justus-Liebig-University Giessen, Feulgenstr. 12, D-35392 Giessen, Germany; 2 European Cystic Fibrosis Society, Karup, Denmark; 3 Department of Respiratory Medicine, University Children Hospital and University of Zürich, Zürich, Switzerland; 4 Interdisciplinary Centre for Clinical Trials (IZKS), University Medical Centre of the Johannes Gutenberg-Universität Mainz, Mainz, Germany; 5 Fundación Española de Fibrosis Quística, Valencia, Spain; 6 Department of Clinical Sciences and Community Health, Dipartimento di Eccellenza 2023-2027, Laboratory of Medical Statistics, Biometry and Epidemiology “G.A. Maccaccaro”, University of Milan, Milan, Italy

**Keywords:** Data accuracy, Data validity, Registries, Epidemiology, Cystic fibrosis, Europe

## Abstract

**Background:**

Patient registries are valuable tools for epidemiological research, especially for rare diseases, and a high level of data quality is essential but not always demonstrated. Although crucial, the quality management process in patient registries rarely includes data validation. The European Cystic Fibrosis Society Patient Registry (ECFSPR) collects clinical data about people with cystic fibrosis (pwCF) in Europe (as defined by the World Health Organisation (WHO) European region). This on-site data validation project was conducted by the ECFSPR to assess feasibility of the project, data accuracy and identify areas for improvement.

**Methods:**

From November 2018 to April 2024 the ECFSPR visited centres to validate data on-site, assessing the accuracy and validity of source data for key variables related to demographics, diagnosis, organ transplant and annual disease progression. We compared data submitted to ECFSPR with medical health records (MHR) at participating centres; standardised variable definitions are used for the ECFSPR data. Accuracy (incl. validity) was expressed as the percentage of validated data points that match the MHR.

**Results:**

We validated source data on-site in 34 of 40 (85%) participating countries and 133 of 397 (34%) centres, for 4024 pwCF (7.5% of the ECFSPR 2021 dataset). Accuracy was high for demographic data (month and year of birth, sex), transplant (> 99%) and annual clinical data on disease progression (selected infections, medication, complications; >94%). Accuracy for genetic information was 96.6% (where the original genotyping laboratory report was available which was for 85% of all pwCF). Anthropometric measurements and lung function data showed lower accuracy (87–88% of the validated data; this was primarily due to non-adherence to the parameters for selection of the encounter for annual lung function assessment. Data for liver disease were also comparatively less accurate (92%); this may reflect diagnostic heterogeneity.

**Conclusions:**

The ECFSPR on-site data validation project demonstrated its feasibility and confirmed the high accuracy of data for critical variables while also revealing specific areas for targeted quality improvement efforts.

**Clinical trial number:**

Not applicable.

**Supplementary Information:**

The online version contains supplementary material available at 10.1186/s13023-025-04153-w.

## Background

Patient registries are critical to reliably describe prevalence, age at diagnosis, genetic diversity, disease burden (including complications), treatment patterns and life expectancy, particularly for rare diseases. Registry data are used to monitor the quality of care and identify areas for improvement, advocate for optimised diagnostic strategies (including newborn screening), improve access to care and therapies, support research publications and facilitate clinical trials. The European Medicines Agency (EMA) [1] and the US Food and Drug Administration (FDA) [2] have both recognized the important role of patient registries in pharmaceutical, post-approval safety and efficacy studies, for which a high level of data quality is essential [3]. The pathway to high quality data is multidimensional: representativeness (the extent to which registry data accurately reflect the target population), completeness (the degree to which all relevant data items are recorded), accuracy (the correspondence between registry data and health records), and validity (adherence of data to definitions) are all elements to be considered.

Cystic fibrosis (CF) is a rare, life-limiting disease affecting approximately 150,000 people worldwide [4]. Since it started in 2003, the European Cystic Fibrosis Society (ECFS) has developed the ECFS Patient Registry (ECFSPR) by integrating data from national registries and individual centres within the WHO European region using harmonised variables, standard inclusion criteria and a common governance structure [5]. The ECFSPR Annual Report (2021), on which the study is based, includes data from 54,000 people with CF (pwCF) across 40 countries and demonstrates high levels of representativeness (self-reported estimated coverage > 80% in almost all countries) and data completeness (97%) [6]. The ECFSPR was qualified by the EMA in 2018 for CF pharmacoepidemiological studies, with timeliness, completeness and accuracy of data identified as critical aspects during the process [7]. Based on these recommendations, the ECFSPR intensified its efforts on data quality and established an on-site data validation project to determine the accuracy of data collection compared to medical health records (MHR). This project also aimed to gain insights into centre- and national-level data collection and documentation processes, enabling targeted support and clarification on data elements as needed. The purpose of this study is to describe the methodology, feasibility, and results of the project to date. We hypothesise that a multinational on-site data validation project will be feasible and confirm an accuracy over 85% for critical data elements.

## Methods

The ECFSPR collects pseudonymized data about CF diagnosis and annual disease progression from pwCF in the WHO European region. In compliance with the European General Data Protection Regulation (GDPR), ethical and data protection approval must be granted by each national authority or centre ethics committee before data can be included in the ECFSPR. Informed consent, including permission for temporary de-pseudonymisation for on-site data validation visits, was obtained from each pwCF or their legal guardian. Harmonised inclusion criteria and standard data coding were established for the ECFSPR data collection and reporting. National registries (NR) [Belarus (BY), Belgium (BE), Czech Republic (CZ), Denmark (DK), France (FR), Germany (DE), Greece (GR), Hungary (HU), Ireland (IE), Italy (IT), the Netherlands (NL), Norway (NO), Russian Federation (RU), Sweden (SE), Türkiye (TR), United Kingdom of Great Britain and Northern Ireland (UK)] upload data to ECFSTracker (Openapp, Dublin, Ireland), a web-based, GDPR-compliant and secure data collection tool, and confirm the conformity of their variable definitions and reporting procedures with those of the ECFSPR. Centres from all other countries [(Albania (AL), Armenia (AM), Austria (AT), Bulgaria (BG), Croatia (HR), Cyprus (CY), Estonia (EE), Finland (FI), Georgia (GE), Iceland (IS), Israel (IL), Latvia (LV), Lithuania (LT), Luxembourg (LU), Republic of Moldavia (MD), North Macedonia (MK), Poland (PL), Portugal (PT), Romania (RO), Serbia (RS), Slovenia (SI), Slovak Republic (SK), Spain (ES), Switzerland (CH), and Ukraine (UA)] enter data directly to ECFSTracker. The ECFSPR has a dedicated, multi-lingual team that provides training and support to users. Data upload and entry include automatic data-entry checks for range, logic, and validity. Completed datasets are submitted to a safe server and ECFSPR statisticians check them for inconsistencies and errors, both cross-sectional and longitudinal. Resulting queries are sent back to data providers to be checked and data are either changed or reconfirmed. In addition, before the annual disease progression dataset is finalised, country reports are sent to a nominated country representative to query for outliers, potential data coding errors in national registries and missing data.

The objectives of the on-site data validation project were to verify, at centre level, the accuracy (degree to which data correctly represents the true values) including validity (defined as adherence to established definitions) of the approved data (the most recent dataset) for a subset of variables related to demographics, diagnosis and annual disease progression. For genetic information, only the original genetic laboratory report was accepted as valid source data. An unpublished CFTR2.org variant list (personal communication, Karen Raraigh) was used as the reference for variant classification. The 16 variables for validation selected (from 141) were chosen because of their importance in disease characterisation and annual disease progression, and relevance to research and epidemiological study trends. They are: age at diagnosis and follow-up, sex, genetic variants (in the cystic fibrosis transmembrane regulator gene), organ transplant status, anthropometry and lung function (height [margin of ± 0.5 cm], weight [margin of ± 0.5 kg], FEV1 [margin of ± 0.1 L]), microbiology (*Pseudomonas aeruginosa*, *Burkholderia cepacia* complex), chronic medications (inhaled antibiotics, pancreatic enzymes, rhDNAse), complications (major haemoptysis, diabetes mellitus, liver disease), and cystic fibrosis transmembrane regulator (CFTR) modulator therapies (lumacaftor/ivacaftor and elexacaftor/tezacaftor/ivacaftor). All countries with centres that input data directly to the ECFSTracker were invited to participate. For countries with their own established national registry and data collection system a different model was proposed, with initial validation visits done together with the NR and the ECFSPR, and subsequently by the NR team alone (Belgium, Czech Republic, France, Germany, Ireland, the Netherlands, and the United Kingdom of Great Britain and Northern Ireland). On-site visits were conducted between 5 November 2018, and 25 April 2024, but suspended between 1 March 2020 and 10 March 2022 due to the COVID-19 pandemic.

The aims of the data quality program were to visit every country in the ECFSPR over a five-year period (approximately 20% of the countries per year) and validate data for at least 10% of the centres in the selected countries, including the CF centre of the nominated country representative, and at least 15–20% of the patients in the country. A one-day visit was planned for centres using direct data entry. Where feasible, centres with more than 50 pwCF were prioritized to maximize the yield of the visits. An additional day was allocated in countries with national CF registries in order to review and understand their data collection and documentation workflows and to gain insight into their national CF software/databases, since these processes are also possible source of anomalies. The percentage of patients selected for validation in each country was stratified by age: 50% aged ≥ 18 years, 40% aged 6–17 years, and 10% aged ≤ 5 years; this reflects the age distribution and age-related disease complexity of CF. A randomly-generated list of maximum 50 pwCF per centre, with accompanying data for the variables to be validated, was generated from the latest ECFSPR database and shared with the centres prior to each visit.

The ECFSPR on-site team (two monitors) signed a confidentiality agreement with each selected centre and ensured that only the MHR of selected pwCF with valid informed consent were reviewed. When necessary to overcome language barriers centre-independent translators or translation software (with appropriate safeguards to ensure privacy through de-identification of information) were employed. For each selected pwCF all variables in the subset used for this study were checked and compared with the latest approved data from the ECFSPR database and categorised as correct, anomalous (incorrect), no data entry, or no source data. The findings were presented and discussed with the centre team at the conclusion of each on-site data validation visit, and a written report (centre and national report) was subsequently sent by the ECFSPR to participants for review and approval within 4 weeks. Inaccurate or inconsistent demographic data, diagnosis and transplant data, but not annual follow-up data, were corrected by the ECFSPR statisticians and these changes were subsequently copied to the user side in the platform. Since 2020, the ECFSPR Annual Report has included a dedicated section on data quality, reporting completeness, and the outcomes of on-site visits conducted in the previous year [6]. The results and lessons learned were shared with the country coordinators at the ECFSPR Steering group meetings from 2019 to 2024 and presented as posters at ECFS conferences in 2019 and 2024.

Clinical data collected from the participating countries were analysed using R v4.0. The degree of data accuracy for each variable was calculated across all validated pwCF and summarised as the median, range, and interquartile range (IQR) across individual countries. To address, bias due to on the heterogenous CF-populations (Figure S1) and validation rates across countries, a sensitivity analysis (Table 2) based on country groups by CF population size (Country Group A: 11000 − 6000 pwCF/ Country Group B: 5999-1000pwCF/ Country Group C: below 1000 pwCF) (Figure S1), including unweighted and weighted analysis, was done and the results compared with the total validated population.

## Results

Between 5 November 2018 and 25 April 2024 (excluding 1 March 2020 to 10 March 2022, due to the COVID-19 pandemic), on-site visits were conducted in 34 of 40 (85%) countries, representing 91% of all pwCF in the ECFSPR in 2021. BY, HU, FI, GE, RU and UA were not visited due to travel restrictions or because informed consent did not permit de-pseudonymisation for data validation.

In the 34 participating countries, 133 of 397 centres (34%) were visited. The median percentage of centres participating across the 34 countries was 50% (range, 13–100%; IQR, 29–100%). In AL, AM, CY, IS, LT, LU, LV, MD and RS only one centre contributed to the ECFSPR at the time of the on-site visit.

Data from a total of 4024 pwCF were validated, representing 8.2% of the participating countries and 7.5% of the pwCF in the entire ECFSPR cohort in 2021. The median percentage of pwCF checked per country was 17% (range, 2–100%; IQR, 9–50%). In countries with fewer than 50 reported pwCF (AL, AM, CY, IS, LT, LU, LV and MD), the entire cohort was validated.

**Fig. 1:**
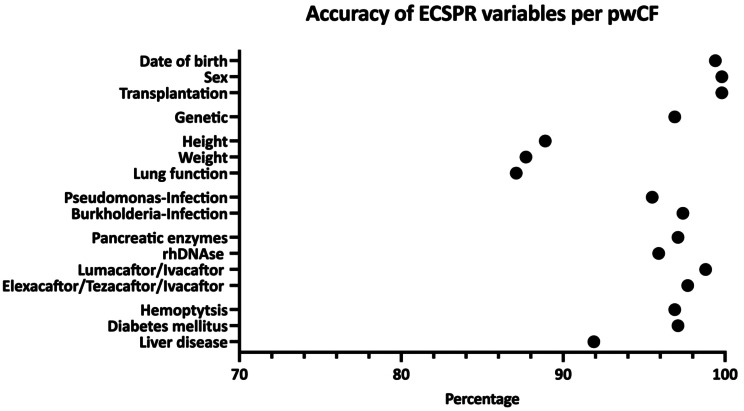
Accuracy of ECFSPR variables per validated people with cystic fibrosis (pwCF). Genetic: accuracy of genetic information based on available original genetic report. Height/Weight/Lung function: accuracy based on valid encounter selection. Lung function (FEV1) only in validated pwCF ≥ 6 years

**Fig. 2:**
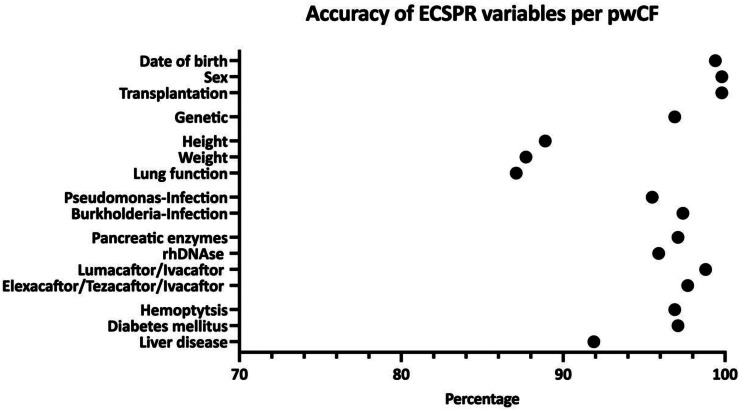
Accuracy of ECFSPR variables per country (median and 25–75% percentiles) Genetic: accuracy of genetic informed based on available original genetic report Height/Weight/Lung function: accuracy based on valid encounter selection Lung function (FEV1) only in validated people with CF ≥ 6 years

Data for month and year of birth, sex, and organ transplant status were accurate for > 99% of validated pwCF (Figs. 1 and 2; Table 1). Source data for genetic information were available for 85.2% of pwCF (country median, 89%; IQR, 77–97%); among those for whom source data were available there were 125 inaccurracies (3.4%; country median 3%; IQR, 1–5%) in the data reported to the ECFSPR, meaning genetic information was accurate for 96.6% of pwCF with an original report.

**Table 1:** Accuracy of ECFSPR variables per all validated people with cystic fibrosis (pwCF) and countries

	pwCF	Countries	
Variable		Median	IQR
Date of birth	99.4%	100%	99–100%
Sex	99.8%	100%	100–100%
Transplantation	99.8%	100%	100–100%
Genetics (report available) Genetics (correct based on report)	85.2% 96.6%	89% 97%	49 – 100% 95–99%
Height	88.9%	89%	83–95%
Weight	87.7%	88%	83–93%
Lung function (FEV1)	87.1%	90%	79–94%
*Pseudomonas -Infection*	95.5%	95%	90–98%
*Burkholderia -Infection*	97.4%	98%	94 – 100%
Pancreatic enzyme	97.1%	98%	96 – 100%
DNAse	95.9%	97%	96 – 100%
Lumacaftor/Ivacaftor	98.8%	100%	94–100%
Elexacaftor/Tezacaftor/Ivacaftor	97.7%	100%	97–100%
Hemoptysis	96.9%	98%	95–100%
Diabetes mellitus	97.1%	98%	94–100%
Liver disease	91.9%	92%	87–96%

Height/Weight/Lung function: accuracy based on valid encounter selection Lung function (FEV1) only in validated pwCF ≥ 6 years Interquartile range (IQR)

Annual follow-up data were accurately collected i.e. in accordance with variable definitions, in > 94% of pwCF for most variables, including chronic medications (inhaled antibiotics, pancreatic enzymes, rhDNAse, lumacaftor/ivacaftor, elexacaftor/tezacaftor/ivacaftor), microbiology (*Pseudomonas aeruginosa* and *Burkholderia cepacia* complex) and complications (diabetes mellitus and major haemoptysis) (Figs. 1 and 2; Table 1). Height, weight, and lung function (forced expiratory volume in 1 s expressed as % of predicted value [FEV1%pred]) followed a complex definition (either the last complete measurement of the calendar year or the corresponding date of the best FEV1%pred during the calendar year), including the selection of the correct date of measurement. These variables were documented accurately and in accordance with the definitions in 88.9% (country median, 89%; range, 38–100%; IQR, 83–95%), 87.7% (country median, 88%; range, 51–100%; IQR, 83–93%), and 87.1% pwCF (country median, 90%; range, 29–100%; IQR, 79–94%) respectively. The accuracy of data on liver disease (including disease severity) was slightly lower (91.9% pwCF; country median, 92%; range, 74–100%; IQR, 87–96%) reflecting different diagnostic assessment strategies.

To address bias due to the heterogenous CF-populations (Figure S1) and validation rates across countries, a sensitivity analysis (Table 2) based on country groups by CF population size (country Group A: 11000 − 6000 pwCF/ country Group B: 5999-1000pwCF/ country Group C: below 1000 pwCF) (Figure S1) including a unweighted and weighted analysis confirmed comparable accuracy rates for most variables. The accuracy of the variables height, weight, lung function in country group A, which contributed 64% of all pwCF in the ECFSPR in 2022, is higher than in the country groups B and C and the weighted analysis showed a higher accuracy for weight/height/FEV1%pred compared to the validated population (92.6%/91.0%/90.5% compared to 88.9%/87.7%/87.1%) (Table 2).

**Table 2:** Sensitivity analysis of accuracy of ECFSPR variables per all validated people with cystic fibrosis (pwCF) divided by countries grouped and weighted by CF populations

Variable	pwCF				
	Country group A *N*=4 C:64% V:30%	Country group B *N*=6 C:22% V:31%	Country group C *N*=24 C:14% V:39%	Countries Weighted per C	All validated
Date of birth	99.8%	99.7%	98.9%	99.7%	99.4%
Sex	100%	100%	100%	100%	99.8%
Transplantation	99.8%	100%	99.7%	99.8%	99.8%
Genetics (report available) Genetics (correct based on report)	**87.1%** **97.3%**	**80.4%** **96.1%**	**87.8%** **96.6%**	**85.7%** **96.9%**	**85.2%** **96.6%**
Height	**96.4%**	**86.6%**	**85.0%**	**92.6%**	**88.9%**
Weight	**94.5%**	**84.8%**	**84.9%**	**91.0%**	**87.7%**
Lung function (FEV1)	**92.6%**	**88.6%**	**83.8%**	**90.5%**	**87.1%**
*Pseudomonas*-Infection	96.1%	94.6%	93.3%	95.4%	95.5%
*Burkholderia*-Infection	98.5%	97.4%	96.5%	98.0%	97.4%
Pancreatic enzyme	97.6%	97.0%	97.0%	97.4%	97.1%
DNAse	96.9%	94.9%	95.9%	96.3%	95.9%
Lumacaftor/Ivacaftor	99.9%	98.8%	98.1%	99.4%	98.8%
Elexacaftor/Tezacaftor/Ivacaftor	97.7%	97.5%	97.8%	97.7%	97.7%
Hemoptysis	98.6%	96.4%	96.1%	97.8%	96.9%
Diabetes mellitus	99.0%	96.0%	96.0%	97.9%	97.1%
Liver disease	96.7%	89.5%	91.1%	94.3%	91.9%

Group A: UK, FR, DE, IT represent 64% of all pwCF and 30% of all validated pwCF Group B: TK, ES, NL, PL, IE, CH represent 22% of all pwCF and 31% of all validated pwCF Group C: Rest of countries represent 14% of all pwCF and 39% of all validated pwCF N: Number of countries C: Percentage of pwCF in the country group divided by total number of pwCF (ECFSPR 2023) V: Percentage of validated pwCF in the countries divided by total number of validated pwCF Weighted: Adjusted analysis to account for the percentage of country group representation

Height/Weight/Lung function: accuracy based on valid encounter selection

Lung function (FEV1) only in validated pwCF ≥ 6 years

## Discussion

The ECFSPR on-site data validation project proved to be feasible and confirmed the high accuracy of critical data elements but also identified areas for improvement.

A high level of data quality is critical to ensure effective use of patient registries. Despite the implementation of centralised quality control measures, data inaccuracies may persist, particularly when more complex variable definitions are used. Source data verification, a mandatory component of quality control in clinical studies, is rarely carried out in patient registries, especially in international collaborations [8] and accuracy and validity are frequently cited as limitations of patient registries [9]. Building upon the nationwide experiences of registries in the United States of America (US) [10], France [11], and the UK [12], the ECFSPR developed its On-site Data Validation Project and has successfully implemented it in 34 of 40 participating countries and 34% of all centres in those countries. The primary challenges encountered were the need for de-pseudonymisation for on-site data validation which required explicit informed consent, and the high number (29) of different languages in the 34 countries. In order to maximise impact within limited budgets the data validation project focused on data elements critical to research and epidemiology, invited all participating countries within a realistic timeframe and established a harmonised joint program with larger national registries.

Data on date of birth, sex, genetic variants and organ transplant status and those on annual disease progression (microbiology results, chronic medications, complications) were accurate in almost all pwCF (> 92%) (Table 1). By contrast, the correct date of measurement for height, weight, and lung function (either of the last complete measurement or the date corresponding to the best FEV1%pred) was not consistently selected resulting in a lower accuracy of 87–89%. A validation project in Switzerland using annual-based documentation and the same variable definitions as the ECFSPR reported slightly lower rates of data accuracy (81–87%) [13]. A French on-site quality audit identified anomalies in 45% of health records and registry entries for FEV1%pred and associated anthropometric measurements, primarily related to the interpretation at the CF centre of the selection rule for the visit with the best FEV1%pred [11]. The integration of a system for the recording of accurate lung function data in a MHRs is critical to obtain an optimal annual overview of all lung function tests. Review of encounter-based documentation, as implemented in the US [10] and the UK (annual review date) [12], has demonstrated higher accuracy: 97.6% for FEV1pred, 96.7% for weight, 92.6% for height, and 95.2% for FEV1%pred. These findings underscore the need for continuous training on the standardised definitions for annual-based documentation of the best FEV1%pred and corresponding weight and height.

Genetic information is critical when it comes to prescribing variant-specific medication. The original genetic laboratory report is the optimal data source and more reliable than other sources, including unverified information copied and pasted to the medical record from referral letters. We found however that the original report was available for only 85% of pwCF and the reported genetic information was inaccurate in 3.4% of cases in which a report was available. These findings are consistent with centre validation reports from Manchester in the UK, which found that original reports were available for 76% of pwCF with an error rate of 6% [14] in the data captured. A US study reported missing genetic information in 5.8% of records for pwCF and inaccurate genetic information in 4.6% of the records for pwCF [10]. Swiss data quality audits found no source data for genetic information in 33% of pwCF and an inaccuracy rate of 0.9% in pwCF with an available genetic report [13]. A French study on compassionate use of therapies reported inaccuracies in the records of 12% of participating pwCF with rare variants [15]. These findings highlight the need for critical review and retrieval of original genetic reports, or retesting for variants, before making genetic-based treatment decisions and also emphasize the importance of the availability to all of a universal and up-to-date genetic variant reference list.

The selection of countries and centres introduced a bias into this multinational quality program. The limitation of a one-day visit, the varying population sizes of the centres and the complexity of the MHR contributed to the heterogeneity in the numbers and percentages of validated pwCF across centres and countries. In countries with a limited number of pwCF, 100% of records were validated, whereas in countries with larger populations, only 2% (UK) or 7% (France/Germany) were validated. The UK CF registry performs audits regularly and for 2018 they reported that records for 506 out of 10,287 pwCF (4.9%) were validated, from 28 of 55 centres (51%) [12]. In-spite of this bias a sensitivity analysis showed consistent accuracy for most variables across three different country groups (grouped by CF population size in the country). For weight/height/FEV1%pred the weighted analysis showed a higher accuracy of 92.6%/91.0%/90.5% compared to 88.9%/87.7%/87.1% for the validated population. It is possible that lessons learned during the project period led to improvements over time in countries that were validated later on in the study. The on-site data validation project will continue to address the GDPR barrier in countries not yet validated, visit new contributing countries and revisit countries with lower rates of valid and accurate data.

## Conclusions

The ECFSPR on-site data validation project has strengthened research collaboration across Europe, enhanced data harmonisation and underscored the need for clear definitions and ongoing training, particularly with regard to variables with complex definitions, as well as demonstrating that such an initiative is possible.

## Electronic supplementary material

Below is the link to the electronic supplementary material.

Supplementary Material 1

## Data Availability

The data that support the findings of this study are available from the ECFSPR; restrictions apply to the availability of the data which were used under license for the current study and are not publicly available. Data can be made available by the authors upon reasonable request with permission from the ECFSPR steering group.
