# Supplementary material for: Data accuracy in the European Cystic Fibrosis Society Patient Registry: results of an on-site data validation project
Source: Orphanet J Rare Dis. 2025 Dec 2;20:622. doi: 10.1186/s13023-025-04153-w (PMC12713276; doi:10.1186/s13023-025-04153-w)
Supplement: Supplementary file 1 — Supplementary Material 1 [file 13023_2025_4153_MOESM1_ESM.docx]

**Figure S1**

**Number of pwCF registered in the ECFSPR in the year 2022 per country and country groups**

adapted from the ECFSPR Annual Report 2022 with permission (6)

= Not in included in the data validation project

**
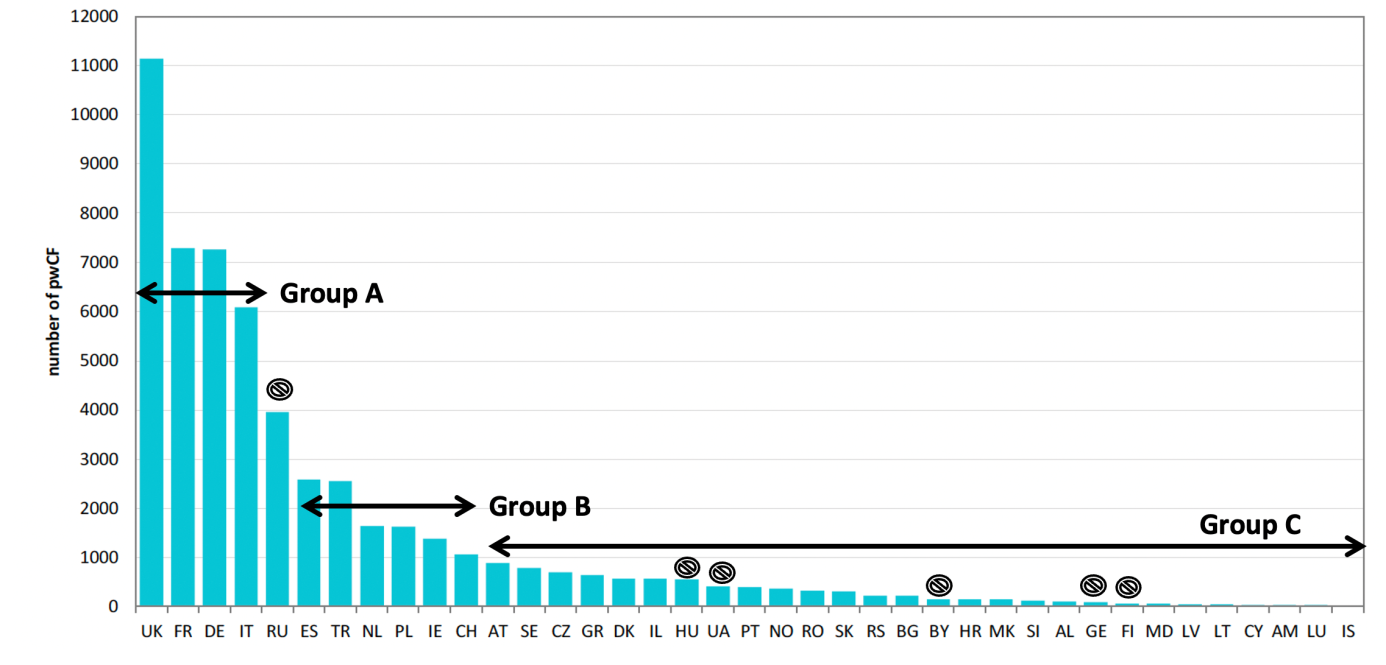
**
